# Beyond the battlefield: The legacy of Archibald McIndoe and the Guinea Pig Club

**DOI:** 10.1016/j.jpra.2026.05.043

**Published:** 2026-05-27

**Authors:** Angelo Federico

**Affiliations:** Corewell Health, 18101 Oakwood Blvd, Dearborn MI, USA

**Keywords:** Reconstruction, History of burn care, Archibald McIndoe, Guinea Pig Club

## Abstract

Originally founded in 1941 during the Second World War, the Guinea Pig Club was a group composed of airmen that sustained severe burn injuries during the war. During this time, the field of reconstructive surgery was still developing and available reconstructive options were extremely limited. Here, we explore the revolutionary advancements in burn care and reconstructive surgery pioneered by Sir Archibald McIndoe, as well as expand on the legacy of the Guinea Pig Club.

## Introduction

The Second World War marked a pivotal period in the evolution of reconstructive surgery. Among the most influential figures of this era was Sir Archibald McIndoe, a pioneering plastic surgeon based out of Queen Victoria Hospital in East Grinstead, England.^1^ Under his care, the Royal Air Force airmen that sustained devastating burn injuries underwent various revolutionary procedures. As these procedures were considered experimental and innovative at the time, the group coined themselves “guinea pigs.”^2^ The work of McIndoe and the Guinea Pig Club not only transformed burn care, but also helped establish core principles of multidisciplinary and patientcentered care that still continue to influence modern plastic surgery today.

### Burn care before McIndoe

Prior to the war, survival from extensive burns was uncommon and reconstructive options were limited. Pre-existing burn care was very limited as well and the main focus of treatment was patient survival. Burn management often relied on tannic acid coagulation, which frequently worsened tissue contracture and directly impaired healing.^3^ Functional and cosmetic outcomes were limited as well, while severe scarring and debilitating contractures were expected. Facial burns often left patients socially isolated and, in some instances, institutionalized. The psychological trauma associated with large burns and the importance of long-term rehabilitation were also not adequately addressed prior to this time period. McIndoe directly challenged the prevailing practices of that time. He abandoned the use of certain toxic topical agents, incorporated saline immersion therapy, encouraged early mobilization of patients, and focused on early reconstructive intervention with split-thickness skin grafting, tube pedicle flaps, and staged reconstruction.^4^

### Surgical innovation and reconstruction

**Fig. 1:**
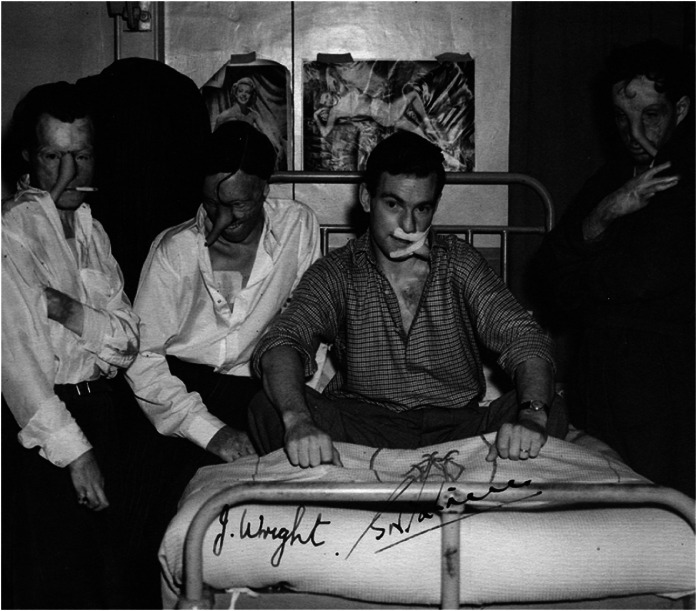
Members of the Guinea Pig Club with tube pedicle flaps at the queen Victoria hospital. Photograph courtesy of east Grinstead museum.

Equally significant to McIndoe’s contribution to burn care was his contribution to reconstructive surgical techniques. Building on principles established by Sir Harold Gillies, McIndoe refined the use of pedicle flaps, staged tissue transfers, and skin grafting to restore both form and function in severely burned airmen.^4^ Many airmen sustained extensive facial burns, resulting in eyelid contractures, nasal deformities, and significant facial scarring. These airmen not only had major functional impairment, but also facial disfigurement. Reconstruction often required multiple staged procedures performed over the course of several years. McIndoe focused on techniques involving tube pedicle flaps and regional tissue transfer to aid in the facial reconstruction of these patients while attempting to preserve the appropriate vascular supply (Fig. 1)*.*^4^ McIndoe also emphasized early operative intervention, which contrasted many prevailing treatment approaches of this period. The reconstructive principles that were used and refined during these procedures helped establish the foundation for many aspects of modern plastic surgery and burn reconstruction. The concepts of staged reconstruction and restoration of function remain central to contemporary reconstruction today.

### Psychosocial rehabilitation and the Guinea Pig Club

The legacy that McIndoe created extends beyond both technical and surgical innovation. His focus on reconstruction involved more than solely surgical repair; it encompassed psychological rehabilitation, social reintegration, and the restoration of personal identity.^5^ He ensured psychological rehabilitation and social reintegration into society were both key components of his patients care. These focuses not only improved patient care and overall outcomes, but also helped foster support from the community. McIndoe’s revolutionary surgical techniques and social initiatives led to the societal acceptance of his burn patients and helped normalize their reintegration into the community. His efforts and holistic philosophy transformed the perception and stigma of burn victims from unfortunate casualties to patients capable of undergoing reconstruction and long-term recovery.2 Because of his efforts, the town of East Grinstead became known as “the town that didn’t stare,” which further supported the community normalization and societal acceptance of burn patients.^2^

### Legacy in modern plastic surgery

The Guinea Pig Club and the work of McIndoe represent a pivotal shift in both surgical philosophy and the history of surgery overall. The wartime reconstructive efforts at East Grinstead not only marked the turning point in which plastic surgery became its own defined surgical specialty, but it provided revolutionary advancements in burn care and helped lay the foundation for modern day reconstructive surgery. The club was founded on core tenets of modern surgery that are still practiced today, including patient-centered care and multidisciplinary collaboration. Many principles now considered central to reconstructive surgery, including multidisciplinary rehabilitation, staged reconstruction, and patient-centered care, can be traced to the treatment philosophy developed around the Guinea Pig Club. The legacy of the club extends beyond technical innovation as it has helped humanize the recovery process and helped shape how modern day surgeons not only operate, but how they approach patients and the overall journey to recovery as well.

## Funding

None.

## Ethical approval

Not required.

## Declaration of competing interest

None declared.
